# Multicentre quality improvement initiative to improve patient education and safety in the prescription of Sodium-Glucose transporter 2 inhibitors

**DOI:** 10.1136/bmjoq-2025-003667

**Published:** 2025-12-10

**Authors:** Lior Saad, Aditya Adiga, Mohamed Anwar Mohamed, Pratyusha Saha, Michael Kuehl, Prithwish Banerjee, Patrick Tran

**Affiliations:** 1 Cardiology Department, University Hospitals Coventry and Warwickshire NHS Trust, Coventry, UK; 2 Warwick Medical School, Coventry, England, UK; 3 Centre for Health & Life Sciences, Coventry University, Coventry, England, UK

**Keywords:** Healthcare quality improvement, Patient education, Patient safety, Quality improvement, Shared decision making

## Abstract

**Background:**

Sodium-Glucose Transporter-2 (SGLT2) inhibitors provide both cardiorenal and metabolic benefits but have several adverse side effects. Effective patient education is critical to ensure safe use and patient compliance. This project aimed to assess and address gaps in patient knowledge about SGLT2-inhibitors.

**Methods and results:**

This quality improvement project was conducted in two tertiary and one district general hospitals in the UK in patients who had been prescribed SGLT2-inhibitors for either diabetes or heart failure. Initially, 100 patients were surveyed on their understanding of SGLT2-inhibitor use, including awareness of indications and side-effects. A patient information leaflet was developed in collaboration with the community pharmacy team and distributed to patients. Six months later, a follow-up survey of 54 patients evaluated their confidence in medication use and knowledge of adverse effects.

The initial survey revealed: 70% were unaware of their medication, 12% had read the manufacturer’s information, 5% were aware of sick-day rules and 12% recognised the risk of UTIs. Diabetic patients demonstrated low awareness of the risk of euglycaemic ketoacidosis (11%) and foot complications (5.6%). Diabetic patients also had higher hospitalisation rates due to drug-related adverse effects. 98% of patients agreed that receiving information about side effects was important. Postintervention, 100% of surveyed patients reported confidence in using SGLT2-inhibitors and knowing when to seek medical advice.

**Conclusion:**

This initiative demonstrates that patients generally lack knowledge regarding the use of SGLT2-inhibitors. Patient education is crucial in improving understanding and medication compliance. Implementing accessible supplemental resources can enhance continued compliance and safety.

**WHAT IS ALREADY KNOWN ON THIS TOPIC**

- Patient information leaflets (PILs) are an important source of medication information but are often lengthy, complex and written above patients’ reading levels. This can reduce understanding, increase anxiety and affect adherence. Despite efforts to simplify PILs, challenges remain in making them both clear and accessible.

**WHAT THIS STUDY ADDS**

- This study demonstrates that a concise, patient-centred leaflet developed with multidisciplinary input can improve understanding and adherence among patients newly started on Sodium-Glucose Transporter-2 inhibitors. Providing clear, relevant information at treatment initiation supports patient confidence and engagement with therapy.

**HOW THIS STUDY MIGHT AFFECT RESEARCH, PRACTICE OR POLICY**

- The study supports the use of co-designed, patient-informed leaflets to enhance medication adherence and understanding. It highlights the need for standardised, health-literate approaches to leaflet development and encourages further research into the broader impact of such materials on treatment outcomes.

## Background

 Sodium-Glucose Transporter 2 Inhibitors (SGLT2-inhibitors), originally developed for type 2 diabetes mellitus (T2DM), are now pivotal in managing heart failure across the entire spectrum of ejection fraction and chronic kidney disease (CKD).^1^,^4^ With prescriptions doubling in the US from roughly 7 to 14 million between 2015 and 2020^5^ and approximately 1.7 million people being eligible for SGLT2-inhibitors in the UK,^6^ their use is surging. A pooled analysis of 10 major randomised placebo-controlled trials further highlights the efficacy of SGLT2-inhibitors.^7^ In heart failure patients, the number needed to treat (NNT) to prevent heart failure hospitalisation over 1 year is 18, and the NNT for total mortality is 76. In CKD patients, the NNT to prevent renal deterioration is 63.^7^

Despite these profound benefits, the safe and effective use of SGLT2-inhibitors hinges on clinicians’ and patients’ awareness of their potential adverse effects. A meta-analysis of randomised controlled trials found that SGLT2-inhibitors were associated with increased risks of genital infection (risk ratio (RR) 3.56), urinary tract infection (UTI; RR 1.06), diabetic ketoacidosis (DKA; RR 2.23) and hypovolaemia (RR 1.14).^8^ Euglycaemic ketoacidosis is another associated risk,^9^ with rarer side effects including perineal necrotising fasciitis, fractures and amputations.^10^ Knowledge of these risks and adherence to ‘sick day rules’ are essential for patient safety and compliance.^11^

### Assessment of problems

Patient information leaflets (PILs) have been shown to help patients in recognising and deciding if they have developed an adverse drug reaction.^12 13^ In the UK, legislation mandates the inclusion of an information leaflet in the medicine box. A national survey of 2897 patients revealed that <50% read the included leaflet, with only 32% reporting that they completely understood it.^14^ Patient focus groups underscore the demand for tailored, accessible information beyond what is already included in the medicine box.^14^

Currently, there is little research investigating patients’ knowledge of the adverse effects associated with SGLT2-inhibitor use, and the effect of written information on patients’ knowledge. This quality improvement initiative aimed to assess baseline patient understanding of the benefits and risks of SGLT2-inhibitor therapy and determine whether a targeted PIL could enhance understanding and confidence, addressing a vital gap in patient-centred care.

## Methods

### Setting

This quality improvement initiative was conducted at University Hospitals Coventry and Warwickshire (UHCW), University Hospital Birmingham NHS Foundation Trust Queen Elizabeth Hospital (both large tertiary teaching hospitals) and Hospital of St. Cross in Rugby (a community hospital under UHCW) from November 2023 to November 2024. The project aimed to improve healthcare delivery and was registered with the trusts’ audit departments. Formal ethical approval was not required, as it involved anonymised feedback surveys and education within routine care for service evaluation.

### Patient population

Eligible patients were adults (≥18 years) with established or newly prescribed SGLT2-inhibitors—with diabetes and/or heart failure being the primary indications—recruited from acute medicine, cardiology and endocrinology departments (both inpatient and outpatient settings). Patients with co-existing severe renal disease (classed as needing either regular dialysis or with a diagnosis of advanced CKD with an estimated glomerular filtration rate (eGFR) <30 mL/min/1.73 m²) were excluded from the trial due to ongoing clinical caution surrounding SGLT2 inhibitor use in advanced CKD. Patient details were anonymised.

### Data collection

In PDSA (Plan, Do, Study, Act) Cycle 1 (November 2023–February 2024), 100 patients were recruited for the initial study to assess baseline understanding of SGLT2-inhibitors. An eight-question survey was developed based on literature review and input from cardiology and pharmacy specialists. It was piloted with 10 patients to ensure clarity and finalised as follows:

1. Do you know why you are being started on this medication?
2. Did you read the PIL that came with this drug?
3. Were you informed of the ‘sick day rules’ when taking SGLT2-inhibitors? (this includes temporarily stopping taking the medication when unwell)
4. Was information given about the risk of developing UTIs, thrush around the vagina/penis and genital infection while taking the medication, and symptoms to be aware of?
5. Were you ever admitted to hospital or seen by a doctor due to the side-effects from taking these medications?
6. *For diabetic patients*—were you warned of the risk of euglycaemic DKA?
7. *For diabetic patients*—were you informed of the risk of lower limb amputation?
8. On a scale of 1–5, how important do you feel it is for patients to know the information provided regarding risks and benefits of SGLT2-inhibitors?

The indication for SGLT-inhibitor and responses to the survey were collected anonymously on a secure Microsoft Forms platform, restricted to the research team within the institution’s Microsoft Teams system.

### Patient information leaflet

**Figure 1:**
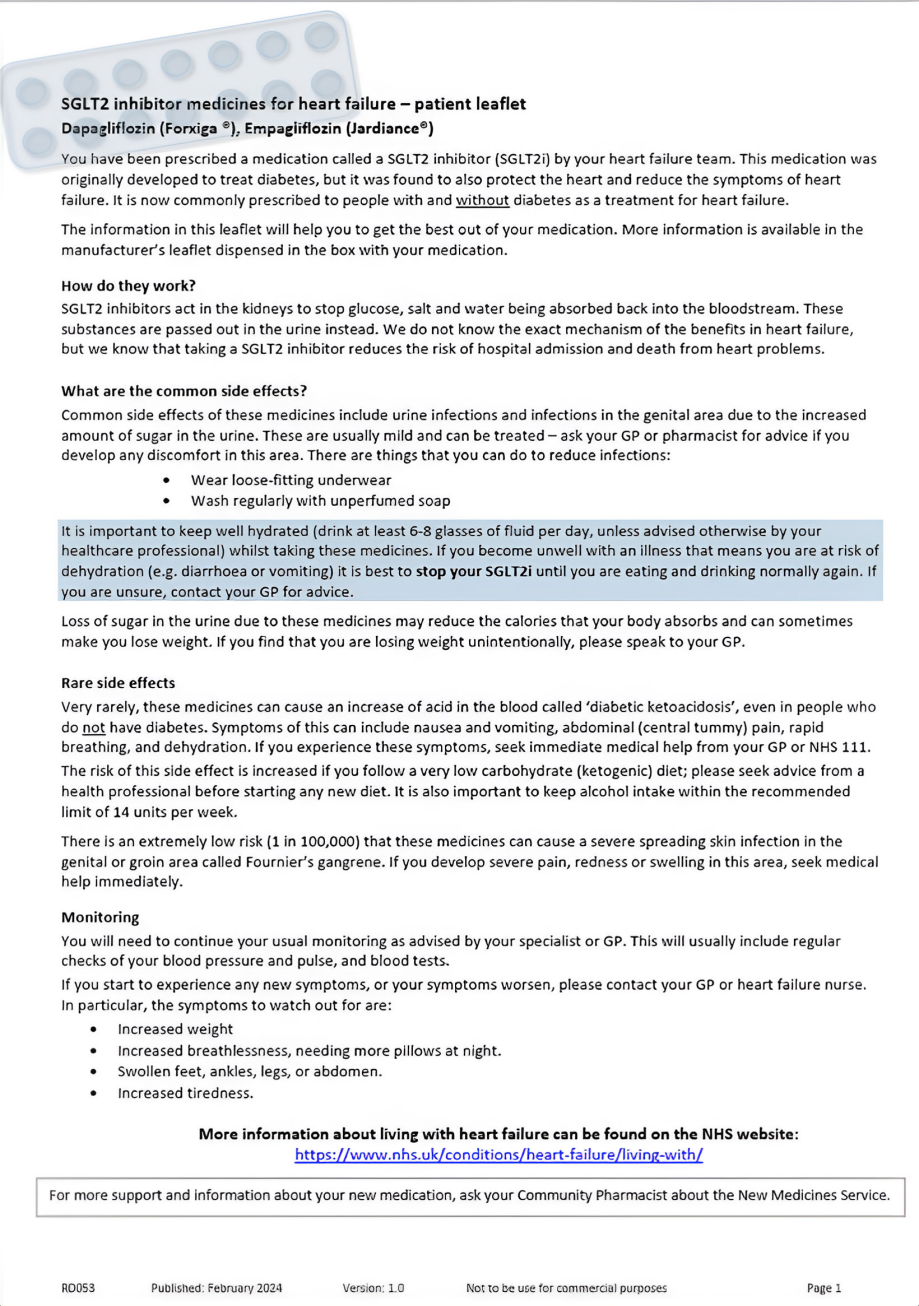
Patient information leaflet created for distribution to patients. GP, General Practitioner; SGLT2, Sodium-Glucose Transporter-2.

The findings from the survey were used to inform the development of a concise PIL in collaboration with the pharmacy department. The leaflet was written in plain English and provided a one-page summary of SGLT2-inhibitor mechanism of action, common and rare side effects, general symptom monitoring and ‘sick day rules’ (see figure 1). Due to significant risk attributed to hypovolaemia and the use of SGLT2 inhibitor, particularly in the context of heart failure and CKD, the leaflet also contained specific information on self-monitoring strategies which included weight checks and identifying symptoms of fluid overload.

The leaflet was made available on the regional formulary website and distributed by the local diabetes and heart failure teams to inpatients and outpatients. It was accessible via a QR code and online link. A 6 month period (March 2024–August 2024) to ensure sufficient time for patients to receive and review the leaflet information and enable consistent dissemination among the speciality teams.

In the PDSA Cycle 2 (August 2024–November 2024), a follow-up survey of 54 patients was conducted to assess the impact on patient understanding of their SGLT2-inhibitor prescriptions and confidence in knowing when to seek further medical advice. This second group of patients constituted a separate, non-overlapping cohort and was analysed independently from the initial study population to validate the findings.

## Results of assessment

### Patient demographics

Among the initial 100 patients surveyed, 75 were male and 25 were female, with an average age of 73±13 years. Fifty-three were prescribed SGLT2 inhibitors for heart failure (16 with preserved ejection fraction) and 47 for T2DM. Dapagliflozin constituted the majority of prescriptions (60%) followed by empagliflozin (40%).

### Baseline knowledge gaps

**Figure 2:**
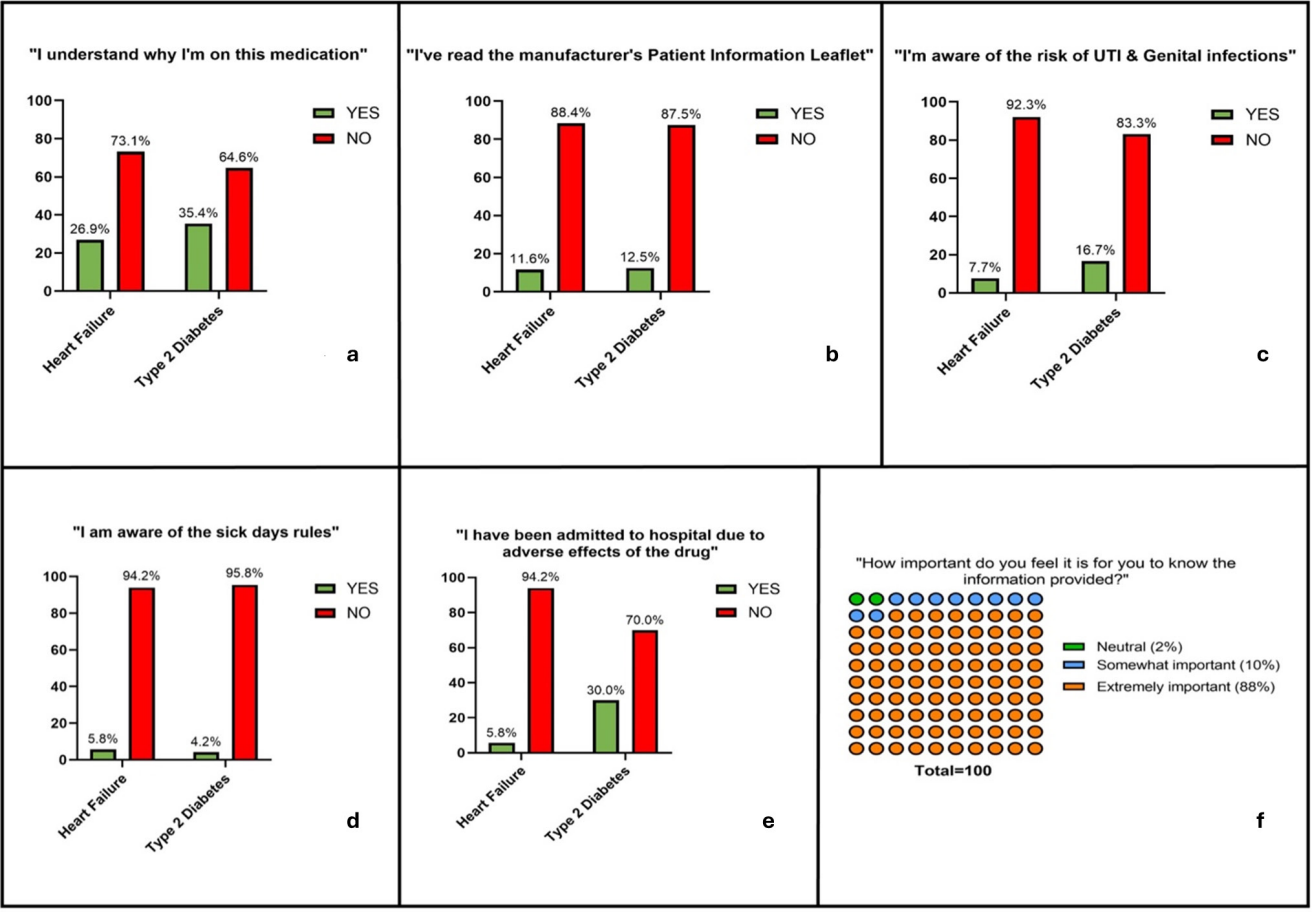
(a–f) Results from initial survey of 100 patients. Responses separated by patients with diabetes and patients with heart failure in **(a–e**). Abbreviations: UTI, urinary tract infection.

In the survey, 69% of patients were unclear of the indication for SGLT2-inhibitors (figure 2a). A slightly higher proportion of patients prescribed SGLT2-inhibitors for diabetes understood why they were taking this medication compared with patients prescribed it for heart failure (35% in diabetic patients vs 27% in heart failure patients). Only 12% of patients had read the originally supplied manufacturer’s PIL (figure 2b). Only 5% of patients were aware of sick-day rules and 12% knew about the risks of UTIs (figure 2c). Among patients on SGLT2 inhibitors for T2DM (n=54), 11% were aware of euglycaemic ketoacidosis risks and 5.6% of potential foot complications risks. Awareness of sick day rules was equally low in both heart failure and T2DM groups (figure 2d). However, reported rates of hospitalisation/seeking medical attention due to adverse effects from SGLT2-inhibitors were more frequent in T2DM patients than heart failure patients (figure 2e). Notably, in both groups of patients, the perception of the importance of being knowledgeable about the risks and benefits of SGLT2-inhibitors was high with 98% of all patients reporting that it was important to receive this information including drug side-effects from their prescribing clinician (figure 2f).

### Postintervention outcomes

In PDSA Cycle 2 (6 months after the development and distribution of the one-page PIL), 54 patients were surveyed (36 male, 18 female). The second group of patients represented an independent cohort, distinct from the initial study population. All patients surveyed had heart failure, equally split among reduced and preserved ejection fraction, and 18 with co-existing T2DM. All (100%) reported improved confidence in taking SGLT2-inhibitors and knowing when to seek medical attention for possible side effects of SGLT2-inhibitors, reflecting a significant improvement postintervention.

## Lessons

To the best of the authors’ knowledge, this quality improvement initiative is the first to explore patients’ awareness and understanding—or a lack thereof—of key information regarding SGLT2-inhibitor therapy, revealing critical knowledge gaps. Its strengths include a robust initial cohort (100 patients including heart failure and T2DM), and the multi-centre design—both of which increase the generalisability of our findings.

### Baseline knowledge deficits

In our initial survey, most patients did not understand why they took SGLT2-inhibitors, and knowledge of side-effects and sick day rules was also poor. Although the exact reasons for this are unclear from our results, it correlates with the general lack of medication knowledge among patients with T2DM and heart failure reported in the literature. In a quantitative study of 108 heart failure patients in France, >50% were unclear whether they had heart failure, and 25% were unsure of the purpose of their heart failure medications.^15^ Qualitative studies from the UK further confirm that many heart failure patients struggle to understand their diagnosis and the purpose of their medications.^16 17^ General medication knowledge in patients with T2DM is also lacking. In a study of 44 American patients with T2DM, the median score achieved on a diabetes medication knowledge questionnaire (testing knowledge of medication name, purpose, side effect etc) was 5 out of 8. None were able to answer all questions correctly.^18^ Interestingly, HbA1c blood levels were inversely associated with knowledge scores, indicating a link between patient knowledge and measurable clinical outcomes.^18^

### Differences in awareness between T2DM and Heart Failure

From the initial survey, 35% of patients taking SGLT2-inhibitors for T2DM understood the medication’s purpose, compared with just 27% of those prescribed for heart failure. This modest difference in awareness may reflect the more intuitive understanding of SGLT2-inhibitors as glucose-lowering agents in T2DM—due to their mechanism of promoting urinary glucose excretion—versus the more complex and less familiar cardiorenal benefits in heart failure management.^19^ Despite the growing evidence base, current utilisation of SGLT2-inhibitors remains below the number of patients eligible for treatment,^20^ with higher prescribing rates among heart failure patients who also have T2DM compared with those without T2DM.^21^ Multiple studies investigating SGLT2-inhibitor prescribing practices have found variations in prescribing behaviour and rates depending on the indication.^22^,^26^ Aligned with our findings, this underscores a clear need for improved patient education, particularly for the heart failure population. Further research is required to understand why patients lack key knowledge of their medication, and what can be done to improve this.

### Limited engagement with standard information leaflet

Only 12% of patients reported reading the information leaflet supplied in the medication box—a figure significantly lower than the 47% reported in a national survey of 2897 UK patients.^14^ This may reflect the limitation of our small sample size, but it also aligns with broader trends showing that many patients overlook the manufacturer’s leaflet. In the aforementioned national study, common reasons include relying on the medical professional to impart key information, practical difficulties in reading or understanding the written information (only 32% reported completely understanding the leaflet).^14^ This correlates with other studies that have shown that PILs provided in medication boxes are often poorly designed and do not cater for older patients in terms of readability or age-specific information.^27 28^

### Value of tailored, concise information leaflets

Our findings showed that 98% of patients valued receiving medication and details of potential side-effects from their prescribing clinician. This is consistent with existing literature, highlighting side-effect information is a common reason for reading PILs^14 29^ and most valued section of a leaflet.^12 30^ Importantly, such information found in leaflets helps patients recognise and decide whether they are experiencing side effects or adverse reactions.^12 13^ Patients clearly value written information that is well-designed, easy to understand and tailored to their individual needs.^31^,^33^ Unlike previous studies focusing on specific conditions and their understanding of related medications, our study is among the first to explore patient understanding specific to SGLT2-inhibitors. After identifying the gaps in patients’ knowledge and what they desired to know, we developed a concise and tailored patient-specific information leaflet. A follow-up survey confirmed that such tailored leaflets are acceptable and effective tools for imparting important medication information. Evidence suggests that well-designed, tailored written information can influence patient behaviour^34^ and potentially improve adherence to diabetic medication.^35^ Further research is required to better understand their long-term impact on knowledge, adherence and clinical outcomes—both for patients taking SGLT2-inhibitors and in general.

### Limitations

Our study has several limitations. Key confounding factors were not collected, such as the duration of SGLT2-inhibitor use. Patients taking these medications for longer periods may have either retained less information or experienced complications/side effects, influencing their responses. We also did not collect data on medication adherence or clinical outcomes; however, the focus of this quality improvement initiative was on general understanding of medication use. Furthermore, it was not always clear if the ‘admissions for side-effects’ were primarily due to the adverse effects of SGLT2-inhibitors, or whether they were due to other factors, based on self-reported information. The absence of data on co-morbidities (such as peripheral vascular disease), and co-prescribed medications further limits interpretation of the data. Our study also did not follow-up with the initial surveyed cohort to seek their opinions on the new leaflet and determine the leaflet’s impact on their medication knowledge. Finally, future studies should investigate larger cohorts across more centres to confirm the generalisability of our findings.

### Implications for the future

Our study demonstrates that patients with diabetes and/or heart failure have limited self-reported understanding of the indications, side-effects and ‘sick day rules’ regarding SGLT2-inhibitor therapy. The tailored PIL, developed through this quality improvement project, addressed these gaps as demonstrated in the follow-up survey with all patients reporting improved confidence in SGLT2-inhibitor use and recognising side effects. Clinicians should adopt similar tailored educational tools into routine care for complex medications like SGLT2-inhibitors, leveraging electronic systems and artificial intelligence such as general practitioner electronic health record platforms like ‘AccuRx’ to deliver tailored messages to a large patient cohort. One example would be to send AI-driven text messages, for example, ‘Dapagliflozin users—pause if unwell with diarrhoea or vomiting’ can help reinforce the ‘sick day rules’. Natural language processing can translate or simplify messages for non-English speaking patients to enhance accessibility.^14 36 37^ These systems may enable more efficient and effective delivery of personalised information.

## Conclusions

This study underscores the importance of improving patient understanding and knowledge surrounding SGLT2-inhibitors through the use of a tailored and concise PIL. As prescribing of this relatively new class of medication increases, ensuring patients are well-informed about side-effects and when to seek medical attention is essential. Patients clearly value receiving this information in a clear and accessible format. While our quality improvement initiative showed promise, further research is necessary to determine whether improved patient education translates into better adherence to SGLT2-inhibitors and fewer adverse-event related hospitalisations. Ultimately, empowering patients through targeted education is a key strategy to optimising therapeutic outcomes for SGLT2-inhibitor therapy.

## Data Availability

Data are available upon reasonable request.
